# Rethinking family planning success metrics: moving beyond coverage

**DOI:** 10.3389/fgwh.2026.1864815

**Published:** 2026-08-17

**Authors:** Franciele Hellwig, Aluisio J. D. Barros

**Affiliations:** 1 International Center for Equity in Health, Federal University of Pelotas, Pelotas, Brazil; 2 Post-graduate Program of Epidemiology, Federal University of Pelotas, Pelotas, Brazil

**Keywords:** contraceptive autonomy, family planning, global healh, national health surveys, sexual and reproductive health and rights (SRHR)

## 1 Introduction

Over the past decades, the global discourse on reproduction has undergone a profound transformation. Early demographic debates were largely centered on reducing high fertility rates, with policy attention focused on population growth and its economic implications. This emphasis gradually shifted toward birth control, prioritizing the expansion of contraceptive methods to allow families to limit or space births to achieve better health and economic outcomes. More recently, the framework has evolved into a comprehensive sexual and reproductive health and rights approach that situates reproductive decision-making within a human rights paradigm, emphasizing bodily autonomy, gender equality, informed choice, and freedom from coercion. As a result, voluntary family planning has been included in several global agendas (1).

In most low- and middle-income countries, substantial investments in family planning have expanded contraceptive availability, increased modern contraceptive prevalence, reduced unmet need, and reduced fertility rates. These gains reflect the success of sustained political commitment, strengthened supply chains, and integration into primary health care (1). However, expansion of access does not automatically translate into women's contraceptive autonomy (2). Prevailing metrics of success remain heavily centered on coverage, demographic, and cost-effectiveness lenses that may not reflect women's lived preferences (3–6). The SDG indicator of demand for family planning satisfied offers a useful global benchmark, yet it tells only part of the story. It overlooks women's own perceived need or desire for contraception, and fails to assess whether the method chosen aligns with women's preferences, whether the decision was free from pressure or bias, or whether they received adequate counseling to make an informed choice (3). Similarly, indicators such as prevalence of contraceptive use and cost per couple-years of protection prioritize uptake and duration over women's choice, satisfaction, and autonomy. This emphasis risks obscuring method skew, provider bias, dissatisfaction with the method used, or discontinuation due to side effects not previously disclosed.

## 2 The neglected signal: distorted method mix

**Figure 1:**
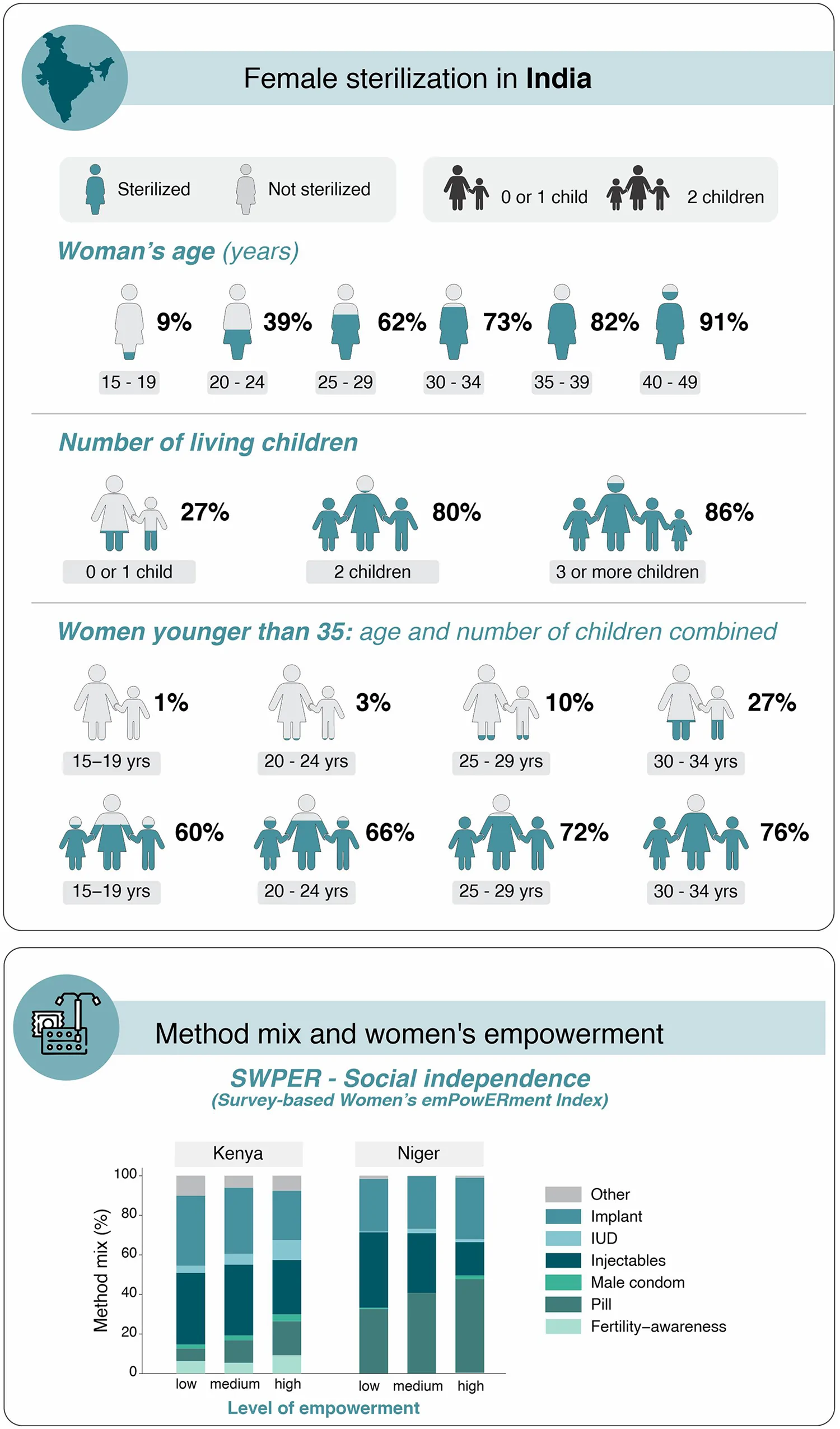
Examples of method skew and variation in contraceptive use across contexts. Note: Data are from the Demographic and Health Surveys conducted in India (2015), Kenya (2022), and Niger (2021). Estimates are derived from previously published research and supplementary analyses. Sources: Hellwig F et al. The role of female permanent contraception in meeting the demand for family planning in low- and middle-income countries. Contraception. 2022; and Hellwig F et al. Women's empowerment and life stage: Assessing intersectional differences in contraceptive method mix in sub-Saharan Africa. Contraception. 2026.

In several countries, high levels of contraceptive use mask markedly distorted method mixes. One of these distortions is characterized by heavy reliance on female sterilization, often even among younger and lower-parity women (7). In India, for instance, more than 1 in 5 modern contraceptive users younger than 35 with no or only one child had already been sterilized (7) (Figure 1). When irreversible methods dominate, it raises concerns about the quality of counseling, the breadth of options meaningfully offered, and the extent to which informed choice is ensured. The choice for female sterilization is strongly influenced by the belief that it is more cost-effective than reversible methods, by the understanding that it has fewer side effects, and by gender norms that associate male sterilization with a threat to virility (8, 9). Altogether, these explanations point to a problematic configuration in which apparent preference for female sterilization may not reflect genuine reproductive self-determination, but structural and normative constraints. Frequent stock-outs of reversible methods, combined with providers' limited training or skill in inserting and managing certain methods, can narrow the options actually available at the point of care. Restricted financial autonomy, limited mobility, and discriminatory gender norms further constrain women's ability to seek or negotiate the method of their choice.

Notably, countries that experienced remarkable progress in family planning coverage have also implemented policies to diversify the contraceptive method mix and invested in provider training to ensure high-quality counseling and provision. However, such investments have been mostly focused on expanding access to implants and injectables (1). While this focus is fully aligned with a cost-effectiveness approach, particularly in resource-constrained settings, it risks undermining investments that would uphold women's autonomy, equity, and informed choice. Such strategies may overlook experiential dimensions such as satisfaction, side effects, method switching, and the quality of counseling. As a result, policies guided primarily by economic efficiency may reinforce method skew and incentivize provider bias, which manifests through practices such as steering women toward specific methods regardless of their preferences, applying age- or parity-based restrictions, gatekeeping access to certain methods based on provider judgment rather than informed choice, and offering inadequate counseling about side effects and alternatives. These dynamics can deepen inequalities in access and advance coverage targets without necessarily strengthening reproductive rights.

Evidence from several African countries illustrates how method mix is associated with the level of women's empowerment. While implants and injectables remain the most widely used methods in many settings, higher levels of women's empowerment are associated with a reduced reliance on these provider-controlled methods and increased use of options that allow greater bodily autonomy and fewer perceived health side effects (10). In Niger, for example, married women with higher social independence were approximately half as likely to rely on injectables and twice as likely to use the pill, while reliance on condoms was about three times higher than among less empowered women. Similarly, in Kenya, highly empowered women showed lower reliance on both implants and injectables and greater use of alternative methods. Compared with less empowered women, they were nearly three times more likely to use IUDs and the pill, and about 40% more likely to rely on fertility-awareness-based methods (Figure 1). Similar patterns are also seen in several sub-Saharan African countries (10). Thus, such shifts in method mix highlight both the transformative role of women's empowerment and the persistent limitations of family planning services in fully supporting autonomous and informed reproductive decision-making.

Skewed method mixes do not, by themselves, prove constrained choice, as they may partly reflect genuine preferences. Yet when paired with provider bias, programmatic incentives, or restrictive gender norms, such skew is a useful signal of structural limitations within sexual and reproductive health services, revealing gaps in equity, information, and autonomy that coverage indicators alone fail to capture.

## 3 The misinterpreted signal: contraceptive discontinuation

Along with contraceptive uptake, we think of programmatic success in family planning as lower discontinuation rates. Contraceptive discontinuation is frequently framed as a sign of failure, often interpreted as a barrier to achieving sustained contraceptive use. The most protective factors for lower discontinuation are providing women with their preferred method and providing adequate counseling on potential side effects and how to deal with them (11). When the healthcare system cannot do it, it is indeed a strong signal of failure. However, this perspective overlooks the complex realities of contraceptive experiences. Dissatisfaction does not always translate into immediate discontinuation, and substantial dissatisfaction may coexist with continued use (12). Many women tolerate methods that do not fully meet their needs due to limited alternatives, inadequate counseling, or barriers to accessing services. In such constrained contexts, continuation alone cannot be assumed to reflect satisfaction or alignment with women's preferences.

At the same time, discontinuation itself should not automatically be interpreted as a negative outcome. Discontinuation rates are usually used to measure how consistently women continue using a specific contraceptive method over time. Therefore, it includes situations where the user stops using contraceptives or switches to another method. When women can stop using a method that causes discomfort or dissatisfaction and transition to another that better fits their needs, discontinuation may represent responsive, person-centered care rather than programmatic failure. Similarly, discontinuation may reflect legitimate changes in women's reproductive intentions or circumstances, such as the desire to become pregnant or periods of sexual inactivity.

From a sexual and reproductive health and rights perspective, the critical question is not whether women continue using a particular method, but whether they are able to start, switch, and discontinue methods according to their preferences and life circumstances. Similar to distorted method mixes, patterns of discontinuation can therefore function as an important signal of the quality and responsiveness of family planning services, revealing whether counseling, method availability, and service environments truly support contraceptive autonomy.

## 4 The missing dimension: contraceptive autonomy

The patterns described above point to a deeper limitation in how family planning programs are currently evaluated. Widely used indicators capture whether women use contraception and for how long, but provide limited insight into whether the methods used genuinely reflect women's preferences, needs, and life circumstances. As a result, high levels of contraceptive use may coexist with constrained choices, inadequate counseling, or limited opportunities to change methods.

Contraceptive autonomy provides a framework for addressing this gap. At its core, it refers to the ability to make a free, full, and informed choice about contraception. Free choice means that the decision of whether to use a method, and which method to use, is made voluntarily, without coercion, pressure, or structural barriers. A full choice requires access to the widest possible range of safe and effective methods, ensuring that options are available rather than merely nominally offered. An informed choice depends on complete, accurate, and unbiased information about all methods, including their benefits, side effects, risks, correct use, and the potential consequences of nonuse (13). Importantly, contraceptive autonomy also encompasses the ability to discontinue or switch methods when preferences or circumstances change.

Estimating autonomy first requires identifying who is considered to need family planning services. Although available measures of need for contraception represented an important advance in family planning metrics, they remain limited. By focusing only on fertility intentions and current contraceptive use, current approaches do not adequately assess whether the woman truly wants or perceives a need for contraception, whether she intends to use it, or whether an acceptable method is realistically available to her (4, 5). Among contraceptive users, existing indicators rarely evaluate whether women are using their preferred method or one adopted under constraint. As a result, existing metrics may overestimate success by equating use with choice and satisfaction, obscuring persistent gaps in reproductive autonomy and rights-based care (3, 12).

Capturing such complex dimensions within standardized questionnaires used in large national health surveys is inherently challenging. Yet, without greater efforts to incorporate autonomy and satisfaction into measurement frameworks, family planning policies risk continuing to prioritize numerical targets over the substantive realization of sexual and reproductive health and rights.

## 5 Discussion

Persistent high fertility rates, distorted method mixes, and patterns of contraceptive discontinuation are often treated as isolated programmatic challenges. Yet taken together, they reveal deeper limitations in how family planning success is currently defined and measured. If family planning policies are to align more closely with sexual and reproductive health and rights principles, the metrics used to evaluate program performance must evolve.

A growing body of work has proposed person-centered alternatives that more directly capture whether contraceptive behavior reflects individual preference. Contraceptive autonomy, for instance, has been piloted in Burkina Faso, using the 17 items recommended to measure informed, free, and full choice (14). Complementing these efforts, related work conducted in Nigeria and Uganda has proposed a broader contraceptive agency framework, along with a corresponding validated scale comprising 15 items grouped into four subscales: beliefs about rights and perceived decision-making control, decision-making self-efficacy, knowledge aligned with preferences, and control over use or nonuse (15, 16). Translating these advances into large-scale, standardized household surveys, however, remains a substantial challenge. Both measures rely on multi-item batteries designed and validated for specific local contexts, making them considerably more burdensome to administer than the questions that underpin conventional indicators. Scaling them to instruments such as the Demographic and Health Surveys or Multiple Indicator Cluster Surveys would require resolving open questions around respondent burden, conceptual translation across languages and cultural settings, and whether such measures can be meaningfully compared across countries with very different contraceptive markets and social norms. Therefore, these full multi-item measures are unlikely to be feasible at scale any time soon and, in consequence, to inform global monitoring and program benchmarks.

A simpler alternative, preference-aligned fertility management (PFM), has also been proposed to assess the degree to which women's contraceptive behavior matches what they say they currently want, taking a neutral stance toward method type and refraining from judging the legitimacy of reasons for nonuse. This measure has since been validated in Nigeria and Uganda (6, 17–19). Because it relies on only a handful of items, PFM is far more feasible to implement at scale than the measures of contraceptive autonomy or agency and already represents a meaningful improvement over the current indicators. However, it does not capture whether a stated preference itself reflects informed choice nor whether that preference was shaped by structural or normative constraints. Given these trade-offs across existing measures, the following points outline what we believe are more incremental, actionable shifts in how family planning success could be measured and incentivized globally.

First, measurement systems should better capture women's perceived need for contraception and explicitly incorporate indicators of contraceptive autonomy, agency, and preference alignment. This includes assessing intentions to use contraception, monitoring informed choice, examining patterns of discontinuation due to dissatisfaction, and assessing whether the method used aligns with women's preferences, accounting for the structural and normative constraints that shape both preferences and the ability to act on them. A pragmatic path forward may involve identifying a reduced set of core items, drawn from these validated constructs, to pilot for inclusion in standardized national health surveys. Even relatively simple additions, if carefully selected and tested for cross-country comparability, could yield valuable insight into women's experiences and preferences, moving beyond coverage to assess whether contraceptive use reflects women's choice or constrained decision-making.

Second, the indicators used to evaluate family planning programs directly shape programmatic incentives and service delivery priorities. When success is primarily measured through uptake and duration of use, evaluation frameworks tend to prioritize expanding coverage, often at the expense of service quality and responsiveness. Incorporating indicators that capture the balance of the contraceptive method mix and monitor levels of method skew would help make visible situations in which programmatic priorities or provider practices limit meaningful choice. Aligning financing mechanisms and program incentives with these indicators could help shift investments toward services that better support autonomous decision-making.

Third, family planning policies should more systematically incorporate life-course perspectives. Contraceptive needs and preferences vary substantially across stages of life and reproductive trajectories, yet most indicators treat women of reproductive age as a single, undifferentiated group. Adolescents, for example, often face distinct barriers related to age, marital status, parity and financial autonomy (20–22). While perimenopausal women's contraceptive needs and risk profiles, shaped by declining fertility, increasing comorbidities, and shifting method suitability, remain comparatively overlooked by stakeholders, researchers, and routine data collection efforts. Age-related gaps that tend to be even more pronounced in countries with lower levels of family planning coverage (23). Indicators that fail to account for these differences risk masking important inequalities in access, family planning counseling, and method suitability across population groups.

Finally, the way indicators are defined can reinforce the conflation of family planning with demographic objectives. In demographically polarized contexts, where concerns about population growth or decline shape public discourse, indicators that focus narrowly on fertility reduction or contraceptive uptake may inadvertently align service delivery with demographic targets rather than with individual preferences. Strengthening rights-based and autonomy-centered indicators, alongside accountability mechanisms, is essential to ensure that family planning programs remain guided by individuals' reproductive intentions rather than broader demographic objectives.

Together, these shifts could move family planning policy beyond a narrow focus on coverage and cost-effectiveness toward an approach that places autonomy, quality of care, and reproductive rights at the center of program success, aligning policies more closely with the principles of sexual and reproductive health and rights they aim to uphold.
